# Super-Insulating Transparent Polyisocyanurate-Polyurethane Aerogels: Analysis of Thermal Conductivity and Mechanical Properties

**DOI:** 10.3390/nano12142409

**Published:** 2022-07-14

**Authors:** Beatriz Merillas, Fernando Villafañe, Miguel Ángel Rodríguez-Pérez

**Affiliations:** 1 Cellular Materials Laboratory (CellMat), Condensed Matter Physics Department, Faculty of Science, University of Valladolid, Campus Miguel Delibes, Paseo de Belén 7, 47011 Valladolid, Spain; 2 GIR MIOMeT-IU Cinquima-Química Inorgánica, Faculty of Science, University of Valladolid, Campus Miguel Delibes, Paseo de Belén 7, 47011 Valladolid, Spain; fernando.villafane@uva.es; 3 BioEcoUVA Research Institute on Bioeconomy, University of Valladolid, 47011 Valladolid, Spain

**Keywords:** polyurethane, polyisocyanurate, aerogels, super-insulation, mechanical properties

## Abstract

A family of transparent polyisocyanurate-polyurethane (PUR-PIR) aerogels with an interesting combination of physical properties were synthesized. First, their textural properties were analyzed aiming to study catalyst influence on the final porous structures and densities. Their thermal conductivities were measured at different temperatures allowing observation of a clear trend relating the initial formulation with the porous structure and reaching values as low as 12 mW/mK, the lowest found in the literature for aerogels based on this polymer matrix. Contributions to thermal conductivity were calculated, improving the understanding of the porous structure-insulating performance relationship. Moreover, their mechanical properties were studied (elastic modulus, stress at different strains and elastic behavior). The aerogels showed tunable stiffness (elastic modulus from 6.32 to 0.13 MPa) by changing the catalyst concentration and significant elasticity. Thus, super-insulating transparent PUR-PIR aerogels with tailored mechanical properties were obtained opening a wide range of potential applications in the energy, building, automotive and aeronautical sectors, among others. The exceptional insulation of silica aerogels was reached at the same time that their general brittleness was improved while keeping good transparency to visible light (85%, 650 nm). Therefore, these aerogels may constitute an alternative to silica aerogels.

## 1. Introduction

Research on super-insulating materials has become a matter of utmost relevance in recent years. Strict building regulations are promoting the search for innovative materials with improved insulating properties [1]. One of the most promising candidates are aerogels, due to their extremely low thermal conductivity in comparison with traditional thermal insulating materials [2,3,4]. Among their remarkably interesting properties are their low weight, high porosity, huge specific surface area, small pore and particle size and significantly low thermal conductivity [5]. Furthermore, some of them present visible-light transparency, which makes them even more singular materials [6,7,8,9]. The combination of these unique features leads to their wide potential applicability in several sectors and, especially, in the building sector [10,11,12].

Since the first aerogels, developed by Kistler in 1931 [13,14], subsequent studies have focused on silica aerogels because of their versatility and incredibly promising properties. An important landmark occurred in 1989 when organic aerogels started to be explored by Pekala [15]. Organic matrixes provide improved synthetic control of the porous structure [16] as well as increased toughness and compression strength in comparison with inorganic aerogels [17,18,19]. For these reasons, organic aerogels have high relevance in the field of nanoporous materials, with numerous works regarding their synthesis and characterization.

The thermal insulating performance of aerogels is one of their most studied properties, and several models have been developed to predict their final insulating capacity [20,21,22]. Regarding aerogels based on polyurethane, Biesmans et al. synthesized the first polyurethane aerogels in 1998, reaching a thermal conductivity of 22 mW/mK at 10 °C for aerogels of 150 kg/m^3^ [23]. Similar thermal conductivity values were reached by Rigacci et al. at room temperature for PU aerogels in 2004 [24]. More recently, different attempts to reduce their thermal conductivities have been carried out [25,26,27], achieving the lowest value of 17 mW/mW at 23 °C by Diascorn et al. [28]. However, due to stringent current insulating requirements, additional improvements on these values would be welcome.

Moreover, the combination of excellent thermal insulation with adequate mechanical [29,30] and optical properties has recently been attracting attention [31,32]. In our previously published works, we synthesized for the very first time PUR-PIR aerogels with tunable optical properties, ranging from opaque to transparent aerogels [33,34]. In the present study, we analyze in depth their mechanical and insulating properties reaching, as far as the authors know, the lowest thermal conductivity values up to now for aerogels based on a PU-PIR matrix. Additionally, the aerogels herein described not only display extremely high insulating performance and transparency, but their mechanical properties are comparable to those obtained by Ratke et al. [35] for RF aerogels, thus showing a wide range of compression modulus, compression strength and high elasticity by adequately tuning the initial formulation. These super-insulating polyurethane-polyisocyanurate aerogels could constitute an interesting alternative for silica aerogels, improving their mechanical strength. Therefore, these materials can have a wide range of potential applications in the energy management, building, automotive and aeronautical sectors.

## 2. Materials and Methods

### 2.1. Materials

Pentaerythritol was used as polyol (ρ = 1.396 g/cm^3^) and was supplied by Alfa Aesar (Thermo Fisher GmbH, Kandel, Germany). The isocyanate was IsoPMDI 92140 (p-MDI) (ρ = 1.23 g/cm^3^), from BASF Polyurethane (Ludwigshafen, Germany). The catalyst commercialized as KOSMOS 75 MEG (potassium octoate) was obtained from Evonik (Essen, Germany). 

All the solvents were provided by Scharlab, S. L. (Barcelona, Spain): acetone (purity > 99.5%), acetonitrile (purity > 99.9%), DMSO (purity > 99.5%) and tetrahydrofurane (purity > 99.5%) stabilized with 250 ppm BHT.

### 2.2. Aerogels Production

The polyurethane-polyisocyanurate aerogel samples were manufactured by a polymerization process occurring in solution, as previously described [33]. A sol was formed by the addition of a pentaerythritol solution (100 g/L in DMSO) to a solution of isocyanate (44 g/L in CH_3_CN 75% vol./THF 35% vol.) keeping a relationship of 0.43 mol polyol/mol isocyanate. A variable amount of catalyst (from 1 to 20 wt.% over the sum of the polyol and isocyanate mass) was added to the blend, and the sol was stirred at 500 rpm for 20 s. After pouring the solution into a plastic mold, gelation started, as the initial viscosity of the mixture increased until the gelation point was reached. Finally, the obtained gel was completely covered with pure acetonitrile during 24 h and then two washing steps (of 24 h) were performed with the same solvent in order to remove the unreacted compounds. Once gels were completely washed, they were dried with supercritical CO_2_ into an autoclaved (40 °C at 100 bar) in a discontinuous way by a number of batches that depended on the number of samples and its volume. Cylindrical aerogels of around 10 mm of height and 38 mm of diameter were produced for thermal conductivity characterization. Cylindrical samples of approximately 8 mm in height and 15 mm in diameter were produced for mechanical characterization.

### 2.3. Characterization Techniques

#### 2.3.1. Density and Porosity

Density was measured by dividing the mass of the obtained aerogels by their geometrical volume, as described in ASTM D1622/D1622M-14 [36]. 

Porosity (*Π*) was calculated by the following equation (Equation (1)):

(1) $$\Pi =\left(1-\rho_{r}\right)*100$$

where *ρ_r_* is the relative density, defined as Equation (2):

(2) $$\rho_{r}=\frac{\rho }{\rho_{s}}$$

where *ρ* is the geometric density and *ρ_s_* the solid density of the polymeric matrix, i.e., 1.17 g/cm^3^ measured by helium pycnometry [33].

#### 2.3.2. Scanning Electron Microscopy

Aerogels were cut with a metal blade and metalized by a sputter coater (EMITECH K575X Sputter Coater, Fall River, MA, USA). The metal employed for the metallization step was iridium to prevent the structure from being altered [37]. A scanning electron microscope (ESEM Scanning Electron Microscope QUANTA 200 FEG, Hillsboro, OR, USA) was used to obtain micrographs of the porous structure.

#### 2.3.3. Specific Surface Area

The specific surface area of the specimens was measured by nitrogen sorption through a Micromeritics (Norcross, GA, USA) ASAP 2020 instrument at the University of Málaga (Spain). Samples were firstly degassed under vacuum for 24 h at 50 °C. The experiments were run at-196 °C in the range P/P_0_ = 0.05–0.30, and the Brunauer-Emmett-Teller (BET) method [38] was employed for the calculations.

#### 2.3.4. Particle and Pore Size

Particle size (ϕ_particle_) was obtained by drawing the particle contour on the SEM micrographs and by using software based on Image J/FIJI (2.0-1.52q, 2019) [39] to determine their diameter. More than 50 particles were analyzed to obtain an average value of each aerogel.

Pore size was measured by the Barrett–Joyner–Halenda (BJH) method by nitrogen sorption [38]. Since capillary condensation occurs when macropores are present, the total pore volume was calculated by the Equation (3):

(3) $$V_{p}=\frac{1}{\rho }-\frac{1}{\rho_{s}}$$

By assuming cylindrical pores, pore size was calculated through by the relationship with their specific surface area, as described in Equation (4):

(4) $$\Phi_{pore\;}=\frac{4V_{p}}{S_{BET}\;}$$

#### 2.3.5. Thermal Conductivity Measurements

Thermal conductivity was measured by employing a thermal heat flow meter model FOX 314 (TA Instruments/LaserComp, Inc. New Castle, DE, USA), which measures according to ASTM C518 [40] and ISO 8301 [41], properly modified and optimized to measure the thermal conductivity of small samples. The active area of the FOX 314 heat flux transducer was 100 × 100 mm^2^. Since the aerogel samples used for these measurements had a diameter approximately. 38 mm, an external heat flux sensor gSKIN^®^ XM 27 9C (greenTEG AG, Rümlang, Switzerland) was used to measure the heat flow through the samples. This heat flow meter was connected to a data logger gSKIN^®^ DLOG-4219 (greenTEG AG, Rümlang, Switzerland) [42]. This combination allowed measuring in the range of ±550 W/m^2^ with a resolution of 0.41 W/m^2^. 

**Figure 1:**
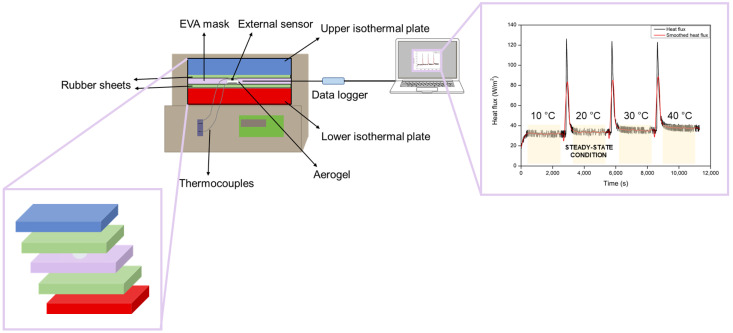
Thermal conductivity setup and heat flux obtained when measuring one sample at four different temperatures.

In order to fill the cavity of the FOX 314 system and avoid convection, EVA foam was used as a mask. This material was located touching the two rubber sheets and the lateral surface of the aerogel sample. This assembly was then placed between two rubber sheets (300 mm × 300 mm × 1.5 mm) for minimizing heat flux fluctuations. The rubber sheets were located in contact with the two plates of the FOX 314, as described in Figure 1. The external sensor was placed between the sample and the upper rubber sheet. Moreover, two thermocouples were added to measure the temperature on both surfaces of the sample during the experiment. The measurements were performed at 10, 20, 30, and 40 °C, keeping a temperature gradient of 20 °C in each case.

The external sensor was calibrated under steady-state conditions as described in ISO 8301 [41]. When measuring at different temperatures, the sensor sensitivity *S(T*) was calculated using Equation (5):

(5) $$S\left(T\right)=S_{0}+\left(T_{s}-22.5\right)\cdot S_{c}$$

where *S_c_* (0.0019 (µV/(W/m^2^))/°C) is the linear correction factor and *S*_0_ is the sensitivity at the calibration temperature (1.55 (µV/(W/m^2^)), 22.5 °C is the calibration temperature, and *T_s_* the mean sensor temperature level. These correction factors were provided by greenTEG AG.

The external sensor provided an output voltage measured every second that was corrected by the sensitivity to obtain the heat flux per unit area (6):

(6) $$q=\frac{U}{S}$$

The obtained heat flux per unit area was smoothed with an adjacent-averaging method by taking 400 data points. Finally, an average of 1200 s was taken from the smoothed signal when the steady state condition was reached (Figure S1). Once the heat flux per unit area was obtained, thermal conductivity could be calculated using Equation (7):

(7) $$\lambda =\frac{q\cdot d}{T_{2}-T_{1}}$$

where *d* is the sample thickness and *T*_2_ − *T*_1_ is the temperature gradient.

#### 2.3.6. Mechanical Tests

Uniaxial compression tests were carried out by using an universal testing machine (Instron model 5500R6025, Norwood, MA, USA) according to ASTM D1621-00 [43]. Tests were performed on cylindrical samples with a diameter to height ratio of approximately 1.8, at ambient conditions (23 ± 2 °C and 50 ± 10% relative humidity as indicated by ISO 291:2005 [44]) and atmospheric pressure. The displacement rate was (height/10) mm/min and a load cell of ca. 1kN. Two different types of experiments were carried out on cylindrical samples of approximately 8 mm height and 15 mm diameter (see Figure S2).

First, five compression-decompression tests were performed using up to a 10% strain and keeping the same speed (height/10) mm/min for both displacements. The energy loss coefficient was obtained from these curves by using Equation (8) [45]:

(8) $$ELC\;\left(\%\right)=\frac{A_{L}-A_{U}}{A_{L}}\cdot 100$$

where *A_L_* and *A_U_* are the areas under the loading and unloading curve, respectively.

In order to ensure good contact between the compression platen and the sample, the elastic modulus (E) was calculated from the linear region of the stress-strain curves of the second compression cycle.

Secondly, compression tests were performed reaching a maximum strain of 75% for all the experiments and employing a preload of around 0.005 MPa. In order to plot the stress (*σ*)-strain (*ε*) curves, these parameters were calculated by using Equations (9) and (10):

(9) $$\sigma =\frac{F}{S}$$

(10) $$\varepsilon =\frac{\Delta h}{h_{0}}$$

where *F* is the applied force, *S* is the surface of the sample cross section, *h*_0_ is the initial height of each sample and *Δh* is the gauge displacement.

The compressive stresses at different strains (*σ*_10%_, *σ*_25%_, *σ*_50%_, *σ*_75%_) were measured.

#### 2.3.7. FT-IR

Infrared spectra were monitored to analyze the characteristic peaks of polyurethane and polyisocyanurate. The spectra of the solid aerogels were collected by an Alpha, Bruker Fourier Transform Infrared spectrometer (Bruker, Billerica, MA, USA) in the range 4000–400 cm^−1^.

First, a background spectrum was deduced from each spectrum. Second, the baseline was corrected. Finally, data were normalized to the asymmetric CH stretching band at 2972 cm^−1^ (whose concentration remains constant). Then, the carbonyl absorptions of the urethane and isocyanurate groups were identified by the second derivative technique and quantified by deconvolution through PeakFit software (v4.12) [46,47].

## 3. Results and Discussion

### 3.1. Aerogel Main Characteristics

The synthesized PUR-PIR aerogels were characterized by measuring their apparent density (*ρ*), relative density (*ρ*_r_), porosity (*Π*), and their porous structure. Values are shown in Table 1.

As demonstrated in our previous work [33], the amount of catalyst employed in each formulation plays a major role on the final properties of the aerogel after the drying step. Densities (between 0.101 and 0.165 g/cm^3^) decrease as the reaction time is shorter, promoting a higher aerogel porosity. This effect on reaction kinetics also affects the porous structure, since particles and pores are larger when formed faster. Nevertheless, when the catalyst concentration is reduced, particles are more effectively packaged, thus giving rise to higher densities and smaller particles and pore sizes.

**Figure 2:**
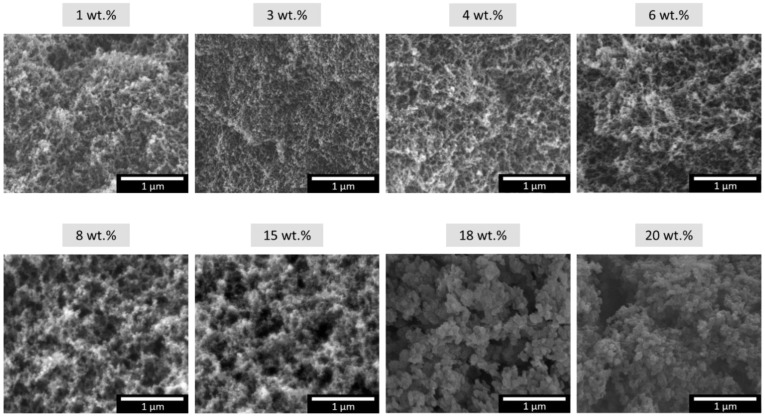
Scanning electron micrographs of aerogels with contents between 1 and 20 wt.%.

These particles have a spherical geometry and are interconnected by chemical bonds. The scanning electron micrographs shown in Figure 2 reveal a 3D open-porous network in which nanometric particles form the solid skeleton. Aerogel particles are composed of a PUR-PIR polymer, and their distribution promotes the generation of small interparticle pores.

The main properties of these samples can be predicted by the SEM images, since the size of the particles clearly increases at higher catalyst contents and, therefore, pores have a larger size. Different packaging depending on particle size was observed, giving rise to the observed changes in the final density values.

The FT-IR spectra of the produced aerogels can be found in the Supporting Information (Figure S3a), as well as the corresponding deconvolution of the amide I region (1610–1760 cm^−1^) (Figure S3c). The peaks corresponding to the main groups—urea from 1642 to 1688 cm^−1^, urethane from 1714 to 1742 cm^−1^ and isocyanurate at 1702 cm^−1^—were identified for all the aerogels.

After peak deconvolution (Table S1), it was observed that the relative area of the peaks associated with urea, urethane and polyisocyanurate were almost constant (Table S1 and Figure S3b) indicating a similar morphology of the polymeric matrix for the materials produced using different catalyst contents.

### 3.2. Thermal Conductivity

#### 3.2.1. Effect of Temperature and Catalyst Content

**Figure 3:**
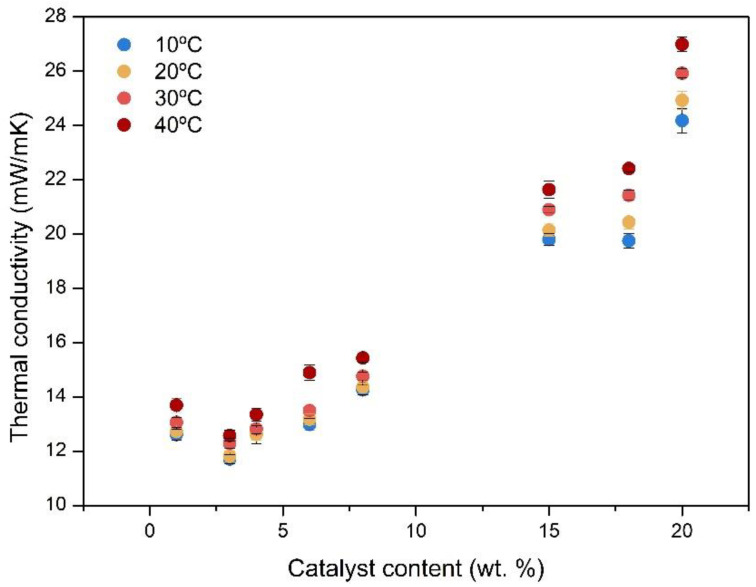
Thermal conductivities of the aerogel samples at 10, 20, 30, and 40 °C.

According to the strong influence that the catalyst has on the structural properties of the final aerogel, notably different thermal conductivities would be expected. Thermal conductivity was measured as explained in the experimental section. The obtained experimental values at four different temperatures (10, 20, 30, and 40 °C) are plotted in Figure 3. Very low thermal conductivities were obtained for all the samples under study, obtaining polyurethane-polyisocyanurate super-insulating aerogels.

A clear trend was detected considering the catalyst amount, lower ratios (1, 3, 4 and 6 wt.%) giving rise to aerogels with the most insulating capacity, with values as low as 12 mW/mK (from 11.71 to 12.99 mW/mK) at 10 °C. As the catalyst amount increased, the thermal conductivity values showed a sharp increase. For 8 wt.% of catalyst, the thermal conductivity was still really low, with a value of 14.26 mW/mK. However, when this amount was further increased, the thermal conductivity reached the highest values of 19.80, 24.61 and 24.18 mW/mK for 15, 18 and 20 wt.% of catalyst, respectively.

When the measurement temperature increased, these values grew slightly, mainly due to radiation contribution to the total conductivity, as analyzed in the following section. Nevertheless, as far as the authors of this work know, the PUR-PIR aerogels herein described show the highest insulating performance reached for polyurethane-based aerogels [23,24,27,28].

#### 3.2.2. Thermal Conductivity Model

Several works have developed thermal conductivity models to predict and evaluate the insulating capacities of aerogels. According to their structures, heat transfer of organic aerogels is controlled by phonon and photon diffusion mechanisms, being described by the addition of the solid (*λ_s_*), gaseous (*λ_g_*) and radiative contributions (*λ_r_*) [48].

(11) $$\lambda =\lambda_{s}+\lambda_{g}+\lambda_{r}$$

In these systems, gas convection within the pores is negligible, owing to their nanometric size.

The solid conduction contribution depends on structural factors such as the backbone structure, particle connectivity, polymer matrix and the aerogel density. Therefore, it can be described generically by Equation (12) [20]:

(12) $$\lambda_{s}=\rho_{r}\cdot \;{\lambda^{\prime }}_{s}\cdot \frac{\upsilon }{\upsilon_{s}}$$

where *ρ_r_* is the relative density, *λ*’*_s_* is the thermal conductivity of the solid matrix and *υ* and *υ*_s_ are the aerogel and solid longitudinal sound speeds.

Regarding the gaseous phase, both the relative amount of gas (porosity) and the thermal conductivity of the gas inside the pores (*λ*′*_s_*) must be taken into account. However, collisions among the gas molecules are minimized due to the nanometric size of the pores present in these samples. Thus, the well-known Knudsen effect takes place promoting a strong decrease of this contribution (Equation (13)) [5,49,50]:

(13) $$\lambda_{g}=\left(1-\rho_{r}\right)\cdot \;{\lambda^{\prime }}_{g}=\left(1-\rho_{r}\right)\cdot \frac{{\lambda^{\prime }}_{g0}\left(T\right)}{1+\frac{2\beta l_{g}}{\phi_{pore}}}$$

where *β* is the correlation factor for the transfer of energy between the gas molecules and the aerogel structure (1.64 for air [51]), *λ*’*_g_*_0_ is the thermal conductivity of the gas inside the pores, *l_g_* is the mean free path of the gas molecules (c.a. 70 nm for air [50,52]), and *φ_pore_* is the average pore size.

Moreover, the radiative contribution is defined by Equation (14):

(14) $$\lambda_{r}=\frac{16\cdot n^{2}\cdot \sigma \cdot T^{3}}{3\cdot (\rho_{r}\cdot K_{s)}}$$

in which *σ* is the Stephan-Boltzmann constant (5.67 × 10^−8^ W/m^2^ K^4^); n is the refractive index (close to 1 for low density aerogels [20]), *T* is the mean temperature; and *K_s_* the extinction coefficient for the solid polymeric matrix (6 × 10^4^ m^−1^ for polyurethane). Finally, the product (*ρ_r_*·*K_s_*) is the extinction coefficient of the aerogel (*K_e_*), assuming that the attenuation mechanisms for infrared radiation is due only to absorption of photons by the polymeric matrix (i.e., scattering of photons is negligible due to the very small particle size in comparison with infrared wavelength).

**Figure 4:**
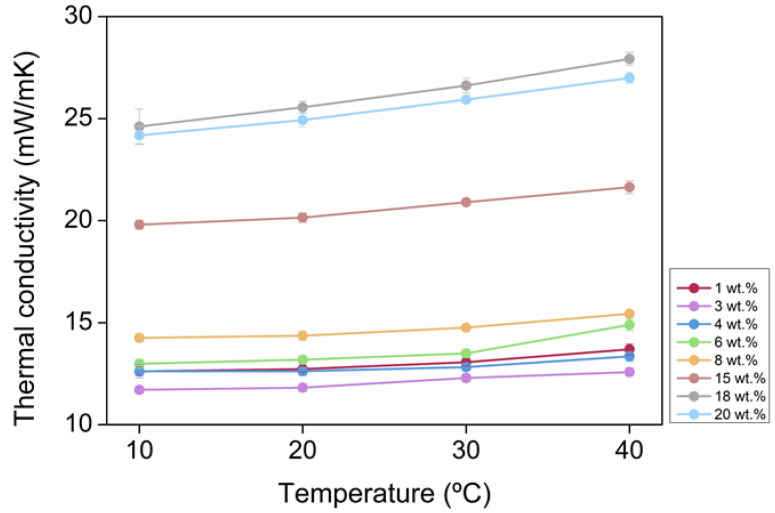
Temperature dependence of thermal conductivity for the PUR-PIR aerogels under study.

According to the previous equations, a clear influence of temperature on the final values of thermal conductivity is expected. A linear tendency of the thermal conductivity with temperature can be observed in Figure 4.

**Figure 5:**
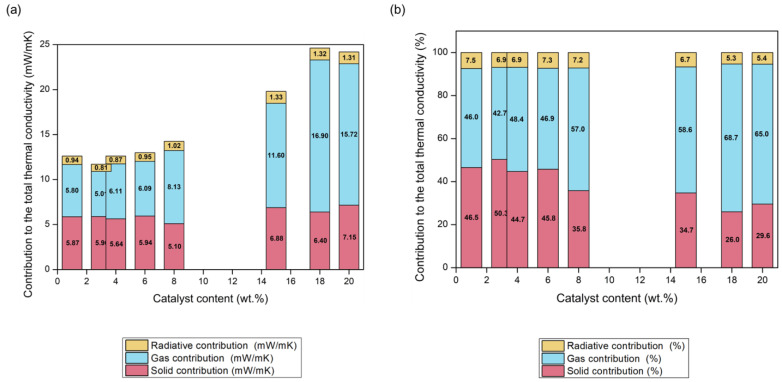
(**a**) Contributions to the total thermal conductivity in mW/mK, (**b**) percentages.

With the aim of assessing the relative weight of each contribution to the total thermal conductivity, gas and radiative contributions were calculated from Equations (13) and (14), respectively. In order to obtain the solid conduction, the other contributions (gas and radiation) were subtracted from the total experimental thermal conductivity. The value of each contribution and the weight of each expressed in percentages are plotted in Figure 5a,b.

It is noticeable that the solid contribution contributes decisively to the final thermal conductivity, being higher than 50% of the total thermal conductivity for some of the samples. Nevertheless, the solid contribution varies from 5 to 7 mW/mK, which is a narrow range. These differences cannot be explained by means of the samples density, because the materials with higher densities show lower solid phase contribution but are based on the phonons transfer through the solid phase. Therefore, when the particles forming the polymeric aerogel are smaller, with an increased surface-to-volume ratio, the size-dependent phononic thermal transport becomes relevant, contributing to a stronger phononic scattering at the boundaries and, thus, a reduction on the solid thermal contribution [53].

Regarding the gas contribution, owing to the nanometric pore size that the PUR-PIR aerogel shows, the Knudsen effect takes place and the gas molecules do not have an effective heat transfer between them, thus reducing the overall thermal conductivity. Conduction through the gas phase increases when the pore size grows, that is, when the amount of catalyst is higher, and the air molecules have a larger path to interact between them. This reached a maximum value of 69% for the sample with an 18 wt.% content of catalyst, showing a difference of ca. 11 mW/mK with the sample of the smallest pores, 1 wt.% (from 16.90 to 5.80 mW/mK, respectively). In fact, the gaseous contribution accounted for 11 of the 12 mW/mK that differentiated the most insulating sample from the poorest one.

Finally, the radiative term did not show a great variation between samples, being in the range from 5.3 to 7.5%, i.e., a difference of around 0.4 mW/mK depending on the final density and the extinction coefficient. The denser aerogels, having a higher polyurethane mass per volume unit, provided more radiation absorbance.

Therefore, the main contribution to the total thermal conductivity was due to gas conduction, followed by solid conduction, depending on the pore size and particle size respectively.

**Figure 6:**
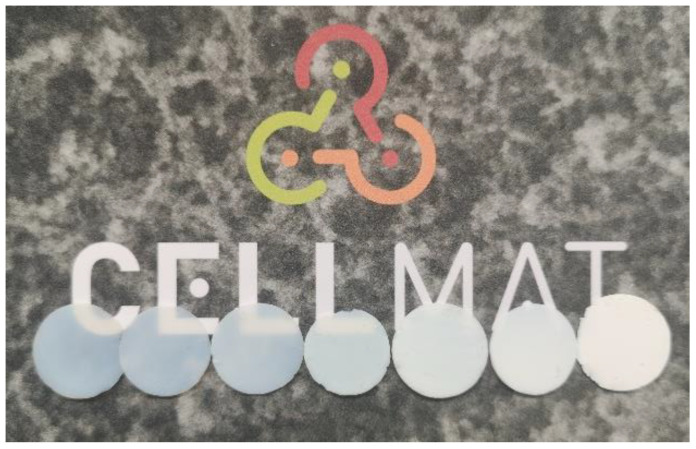
Photograph of the aerogels under study. From left to right: 1, 3, 4, 6, 8, 15 and 20 wt.% of catalyst content.

The combination of these low thermal conductivities with the light transmittance that these aerogels show (studied in detail in [34]) opens a wide range of possible applications such as solar collectors, windows glazing or any application in which insulation and transparency are needed. The transmittance values at different wavelengths for the sample produced with 1 wt.% of catalyst and 1 mm of thickness were 85% for 650 nm, 71% for 532 nm and 51% for 450 nm. Figure 6 shows optical images of the samples proving the transparency of those samples produced with a low catalyst content.

#### 3.2.3. Thermal Conductivity Dependence with the Porous Structure

As has been previously analyzed, catalyst concentration is the main factor controlling the inner structure of PUR-PIR aerogels. Scanning electron micrographs show how the aerogel structures are composed by interconnected spherical particles. These particles present different sizes between samples, leading to a wide range of pore sizes. In this section, different structural parameters affecting the final insulating capacity of the aerogels are discussed.

**Figure 7:**
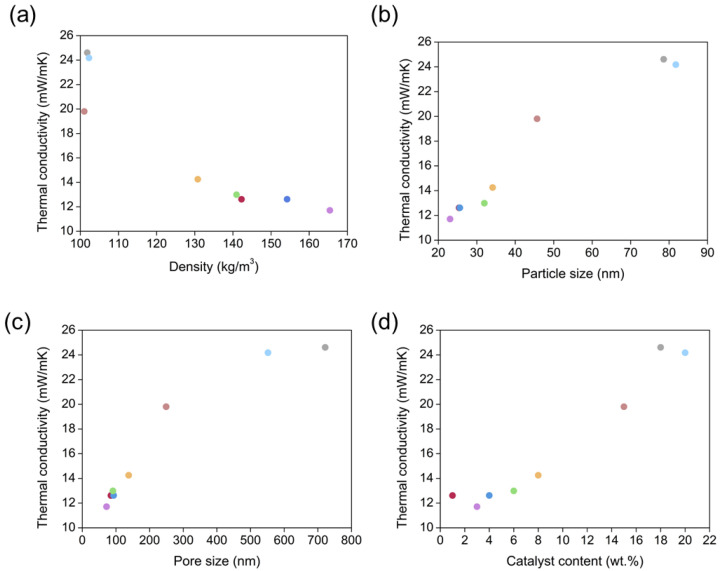
Thermal conductivity at 10 °C as a function of different aerogel properties: (**a**) density, (**b**) particle size, (**c**) pore size, (**d**) catalyst content.

Theoretically, we should expect that an increase in the final density for materials with a similar internal structure would lead to an increment in the proportion of solid network and, therefore, it would promote a structure with a higher number of contacts per particle, i.e., more interconnections than for less dense counterparts. This would lead to a lower thermal resistance and a higher thermal conductivity [48]. However, in our materials there was a relevant dependence between density and the particle and pore sizes. In this way, aerogels with higher densities contained the smallest particles, with the previously mentioned size-dependent phononic thermal transport. Consequently, aerogels with a higher density would present a poorest phononic transfer (stronger phononic scattering), thus reducing the solid contribution to the effective thermal conductivity. Additionally, this increase in the final density leads to a reduced particle size and to a more effectively particle packaging, which notably reduces the average pore size, contributing to decrease the gaseous contribution. In addition, the radiative heat transfer becomes lower when density increases (Equation (14)). This combination of effects is reflected in Figure 7a, which shows the higher the final density, the smaller the total thermal conductivity. This inversely proportional relationship explains why aerogels having densities between 140 and 165 kg/m^3^ show the lowest values of thermal conductivity (between 11.71 and 12.99 mW/mK). As density decreases, the insulating capacity sharply increases, reaching values of 24.18 mW/mK for 102.3 kg/m^3^.

Figure 7b,c display the dependence of thermal conductivity with particle and pore size, respectively. In both cases, the decline in the size of these features gives rise to a remarkable reduction of the conduction through the solid (particles) or gas (pores) phases. The latter contributes to the Knudsen effect appearance by the restriction of gas molecule movement. The graphs suggest that the optimum particle size for obtaining effective insulating PUR-PIR aerogels (*λ* < 20 mW/mK) should be lower than 40 nm, whereas the optimum pore size in order to achieve these ultralow values would be below 200 nm.

Additionally, the relationship between thermal conductivity and the catalyst concentration on each formulation is plotted in Figure 7d. As expected, a clear trend can be seen, since catalyst concentration controls the micro- and macroscopic properties of these aerogels. Thereby, aerogels with a low catalyst content, displaying the highest density and smaller particles and pores, reach the most insulating capacities. In this way, aerogels made with catalyst concentration of 1, 3, 4 and 6 wt % reach thermal conductivity values below 13 mW/mK, whereas the aerogels with a higher content (15, 18 and 20 wt.%) present values above 20 mW/mK. It should be pointed out that all the PUR-PIR aerogels herein described are super-insulating aerogels, and the obtained experimental values provide a significant improvement of the insulating performance compared to those found in the literature for these organic aerogels.

### 3.3. Mechanical Properties

Compression uniaxial tests were carried out in order to evaluate the mechanical behavior of the PUR-PIR aerogels and are discussed in this section.

#### 3.3.1. Elastic Modulus

**Figure 8:**
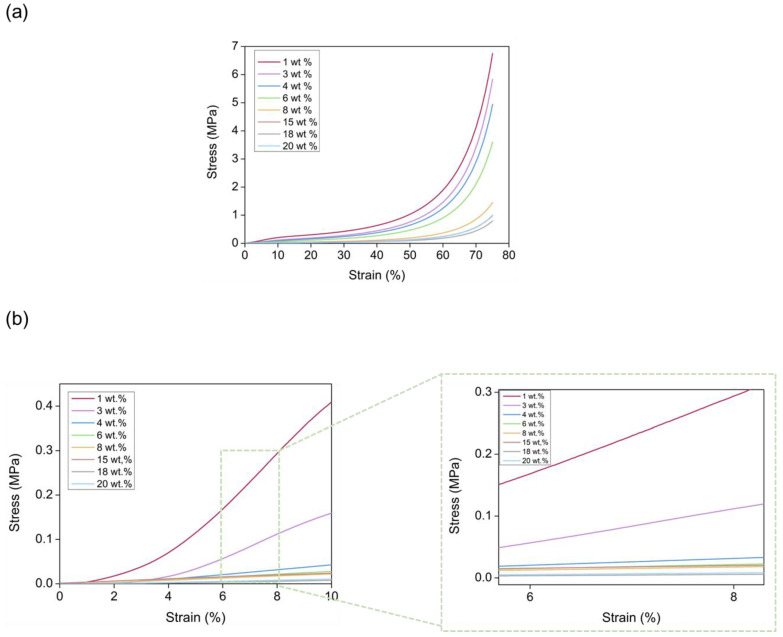
(**a**) Stress-strain curves for the PUR-PIR aerogels until a deformation of 75%. (**b**) Stress-strain curves from the second cycle at a strain of 10%.

Stress-strain curves were obtained for all the aerogel samples containing different catalyst amounts until reaching a strain of 75% (Figure 8a). At low deformations, a linear region was observed where aerogels showed an elastic and reversible deformation. Then, a second region where deformation presented a plastic and irreversible behavior until reaching the final strain was detected. It is noticeable that no breaking point could be found, since these aerogels were densified with increasingly stress, leading to almost plain disks (Figure S2).

**Figure 9:**
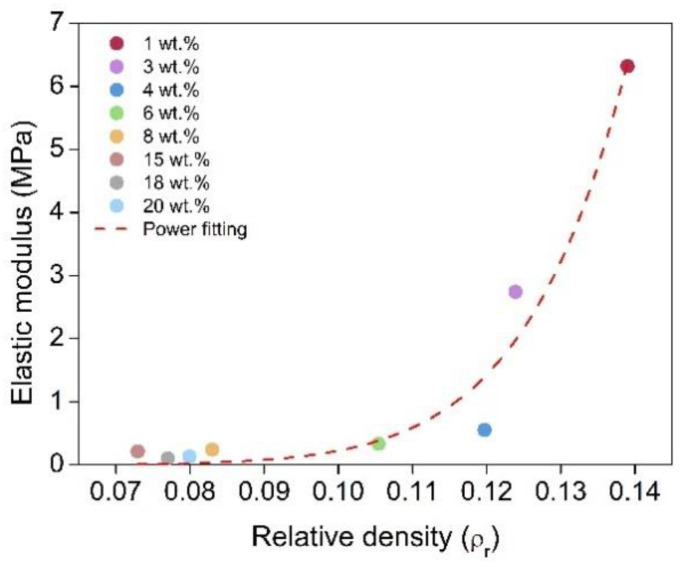
Elastic modulus dependence on relative density and the power fitting.

The corresponding elastic modulus (E) was calculated from the slope of the linear region of the compression until 10% (second cycle) of strain, to improve the measurement accuracy (Figure 8b). There was a clear relationship between the elastic modulus and the catalyst content, the former being reduced as the catalyst amount increases (Figure 9). The sample with 1 wt.% of catalyst reached a maximum value of 6.3 MPa followed by a value of 2.74 MPa for the sample with 3 wt.% of catalyst, whereas it dropped to 0.13 MPa for the sample with the highest concentration of 20 wt.% (numerical values can be found in Table S2). These compressive moduli are similar to those described for other organic aerogels, such as the RF aerogels obtained by Ratke et al. [35]. Thus, the lower the catalyst amount, the higher the stress to be applied for reaching a specific strain.

Due to the influence that density presents on the aerogel’s stiffness, the relationship between density and the Elastic modulus was studied. The power of fitting was obtained by plotting E vs. *ρ_r_* (Figure 9). A value of 10.2 was obtained (Table S3), which is similar to those that have been found in the literature for polyurethane aerogels [25,30]. Clearly, our PUR-PIR aerogels showed a huge range between their elastic modulus values (a 48-fold difference), whereas the difference between their densities was less than two times. Therefore, although there was a clear trend as a function of density, part of this strong increase in stiffness for the materials with lower particle and pore sizes was probably due to the stiffer structure obtained when the catalyst content was reduced.

#### 3.3.2. Stress at Different Strains

**Figure 10:**
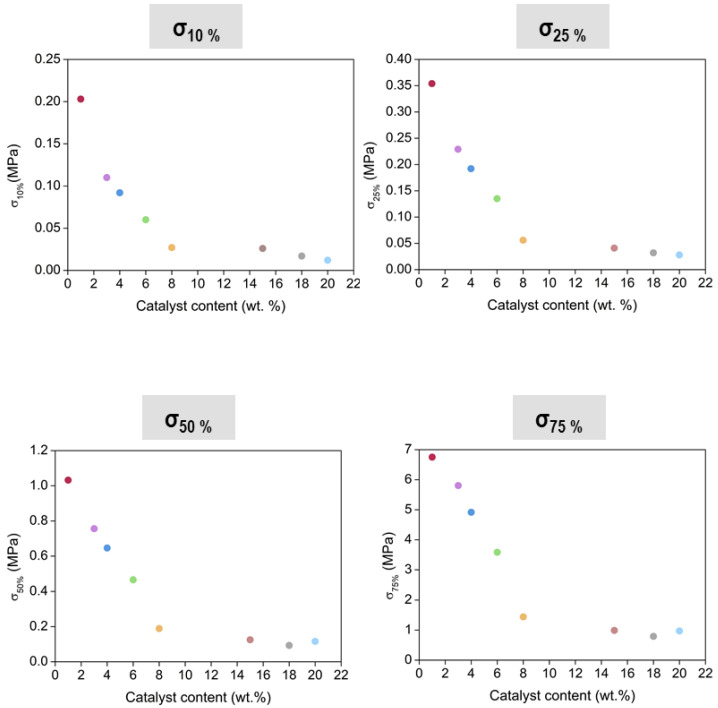
Stress at different strains (10, 25, 50 and 75%) for all aerogel samples.

With data from the same compression experiment, and according to Figure 8, the stress applied for obtaining different strains could be extracted. In this way, the stress needed to deform different percentages could be compared for all the PUR-PIR aerogel samples. Figure 10 displays the numerical values of stress plotted for strains of 10, 25, 50 and 75%. The data indicate how the evolution of the compressive stress seems to be significantly influenced by the variation of the catalyst content and, as a consequence, by the density and by the porous structure. In agreement with the elastic modulus results obtained for all the analyzed strains, as the aerogel had a lower amount of catalyst (leading to smaller particles and pores), the necessary stress for deforming a specific value was higher, and finally it gradually decreased as the concentration increased. These results indicate that PUR-PIR aerogels with tunable mechanical properties can be obtained by adequately modifying the catalyst amount included into the formulation.

#### 3.3.3. Elastic Behavior

**Figure 11:**
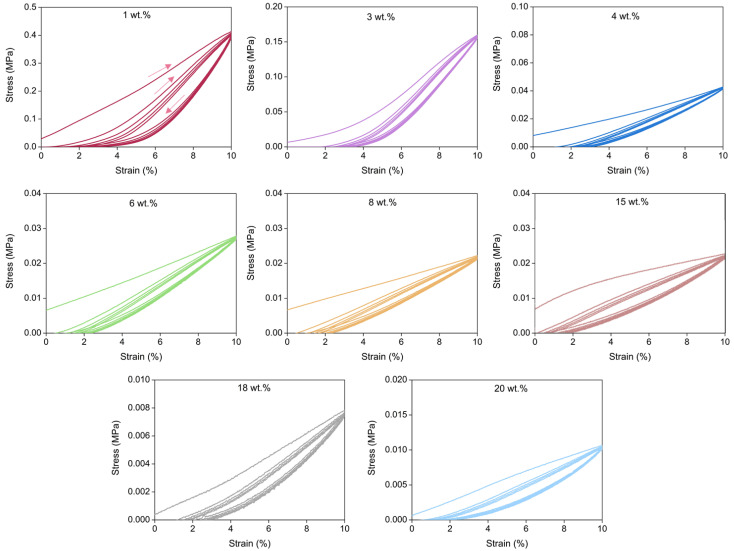
Stress-strain curves for the PUR-PIR aerogels obtained by compression-decompression tests.

An additional set of uniaxial compression experiments was carried out to study the elastic behavior of the PUR-PIR aerogels. By submitting the aerogels to five compression-decompression cycles, their recovery capacity after loading was measured. The recovery capability was evaluated for a deformation of 10%, and the results of these experiments for all the analyzed materials are collected in Figure 11. All the formulations displayed an elastic behavior with an almost complete recovery after the five loading applied cycles. The non-recovered deformation as approximately. 2% for all materials. These figures reflect how the first cycle required a higher stress than the others. However, these results confirm that all the samples had high recovery ratios, displaying a significant elasticity which may be promoted by the presence of the cross-linking nodes inherent to the isocyanurate ring present in the molecular structure of the aerogels [54].

**Figure 12:**
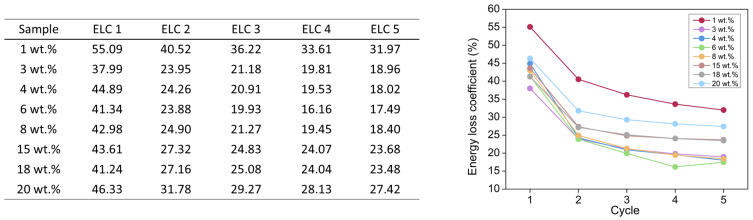
Numerical values for the energy loss coefficient at every compression-decompression cycle and for l the PUR-PIR aerogel formulations and their corresponding graph.

By assessing the hysteresis area, the energy loss coefficient can be calculated (Equation (8)). The numerical values and their graphical representation are displayed in Figure 12.

The ELC was notably higher for the first cycle, whereas it was almost constant during the rest of cycles. Thus, during the first compressive cycle a larger amount of energy was dissipated and, therefore, aerogels were less elastic at this initial point. However, this coefficient decreased as the other cycles were performed, indicating that the aerogels became more elastic. There was not a clear trend with the catalyst amount, although the aerogel with the lowest content showed the highest ELC, meaning that this formulation provided a higher energy dissipation and, as previously discussed, it was the least elastic and most rigid aerogel.

## 4. Conclusions

Transparent polyurethane-polyisocyanurate aerogels with different catalyst concentrations were synthesized and their insulating capacity and mechanical properties were assessed in detail. 

Thermal conductivities were measured at different temperatures (10, 20, 30 and 40 °C). A noticeable trend with catalyst content was observed, as higher thermal conductivities were obtained when increasing the catalyst amount. All the samples showed a superinsulating performance, reaching values from 12 mW/mK for the lowest catalyzed formulation to 24 mW/mK for the highest at 10 °C. The thermal conductivity of the formulation with a catalyst content of 1 wt.% was the lowest value found in the literature for PUR-PIR aerogels. This sample also showed a high value of light transmittance ca. 85% (650 nm) [34] which, in combination with its super-insulating performance, provides a great potential interest. 

The thermal insulating capacity-porous structure features relationship was analyzed, concluding that the reduction of both, pore and particle size, has a stronger influence than the increase in the final bulk density, which occurs as the catalyst content is reduced. It was demonstrated that most of the thermal conductivity reduction was due to the reduction of the gas contribution as a consequence of the Knudsen effect. In addition, the solid contribution was also reduced for the aerogels with lower particle and pore sizes, even those with higher densities. This is attributed to a size-effect that promotes a more intense phonon’s scattering. The radiative heat transfer depending on density did not significantly change with a contribution of ca. 1 mW/mK for all the aerogels.

Compressive mechanical properties were also analyzed. Elastic modulus showed values from 0.13 to 6.32 MPa, resulting in stiffer aerogels to softer ones when the catalyst amount was reduced. This strong difference in the mechanical performance can be explained because of a density increase and stiffer porous structure for the materials produced when using lower contents of catalyst, since their particles and pores have a smaller size.

The elastic behavior of the synthesized aerogels was assessed by performing five compression-decompression cycles, all of them showing a high elasticity and recovery ratios for 10% of strain.

## Figures and Tables

**Table 1:** Aerogel main characteristics.

Catalyst Amount (wt.%)	*ρ* (g/cm^3^)	*ρ_r_*	*Π* (%)	*ϕ*_particle_ (nm)	*ϕ*_pore_ (nm)
1	0.142	0.12	87.86	25.40 ± 6.46	85.04
3	0.165	0.14	85.89	23.04 ± 6.01	72.08
4	0.154	0.13	86.84	25.58 ± 6.39	93.05
6	0.141	0.12	87.97	31.96 ± 6.93	91.09
8	0.131	0.11	88.84	34.12 ± 7.79	137.85
15	0.101	0.09	91.38	45.67 ± 12.26	249.14
18	0.102	0.09	91.31	78.60 ± 15.97	721.94
20	0.102	0.09	91.27	81.76 ± 18.73	551.94

## Data Availability

Not applicable.

## Supplementary Materials

The following supporting information can be downloaded at: https://www.mdpi.com/article/10.3390/nano12142409/s1, Figure S1. Heat flux obtained with an external sensor for different measurement temperatures under the sta-tionary method, Figure S2. Aerogel PUR-PIR samples before (left) and after (right) the uniaxial compression tests, Figure S3. (a) FT-IR spectra of some PUR-PIR aerogels, (b) Relative area of each of the products of the amide I region for the different samples, (c) Example of an FT-IR spectrum deconvolution, Table S1. Urea, urethane and isocyanurate total and relative areas, Table S2. Elastic modulus value for all the aerogel samples, Table S3. Fitting parameters for the elastic modulus vs. the relative densities of the PUR-PIR aerogels.
