# Supplementary material for: Super-Insulating Transparent Polyisocyanurate-Polyurethane Aerogels: Analysis of Thermal Conductivity and Mechanical Properties
Source: Nanomaterials (Basel). 2022 Jul 14;12(14):2409. doi: 10.3390/nano12142409 (PMC9320143; doi:10.3390/nano12142409)
Supplement: Supplementary file 1 [file nanomaterials-12-02409-s001.zip › nanomaterials-1789290-supplementary.pdf]

nanomaterials

MDPI

Supplementary Material

Super-Insulating Transparent Polyisocyanurate-Polyurethane Aerogels: Analysis of Thermal Conductivity and Mechanical Properties

Beatriz Merillas [1,*], Fernando Villafañe [2] and Miguel Ángel Rodríguez-Pérez [1,3,*]

[1] Cellular Materials Laboratory (CellMat), Condensed Matter Physics Department, Faculty of Science, University of Valladolid, Campus Miguel Delibes, Paseo de Belén 7, 47011 Valladolid, Spain
[2] GIR MIOMeT-IU Cinquima-Química Inorgánica, Faculty of Science, University of Valladolid, Campus Miguel Delibes, Paseo de Belén 7, 47011 Valladolid, Spain; fernando.villafane@uva.es
[3] BioEcoUVA Research Institute on Bioeconomy, University of Valladolid, Spain
* Correspondence: b.merillas@fmc.uva.es (B.M.); marrod@fmc.uva.es (M.Á.R.-P.); Tlf.: +34-983-42-31-94 (B.M.); Tlf: +34-983-18-40-35 (M.Á.R.-P.)

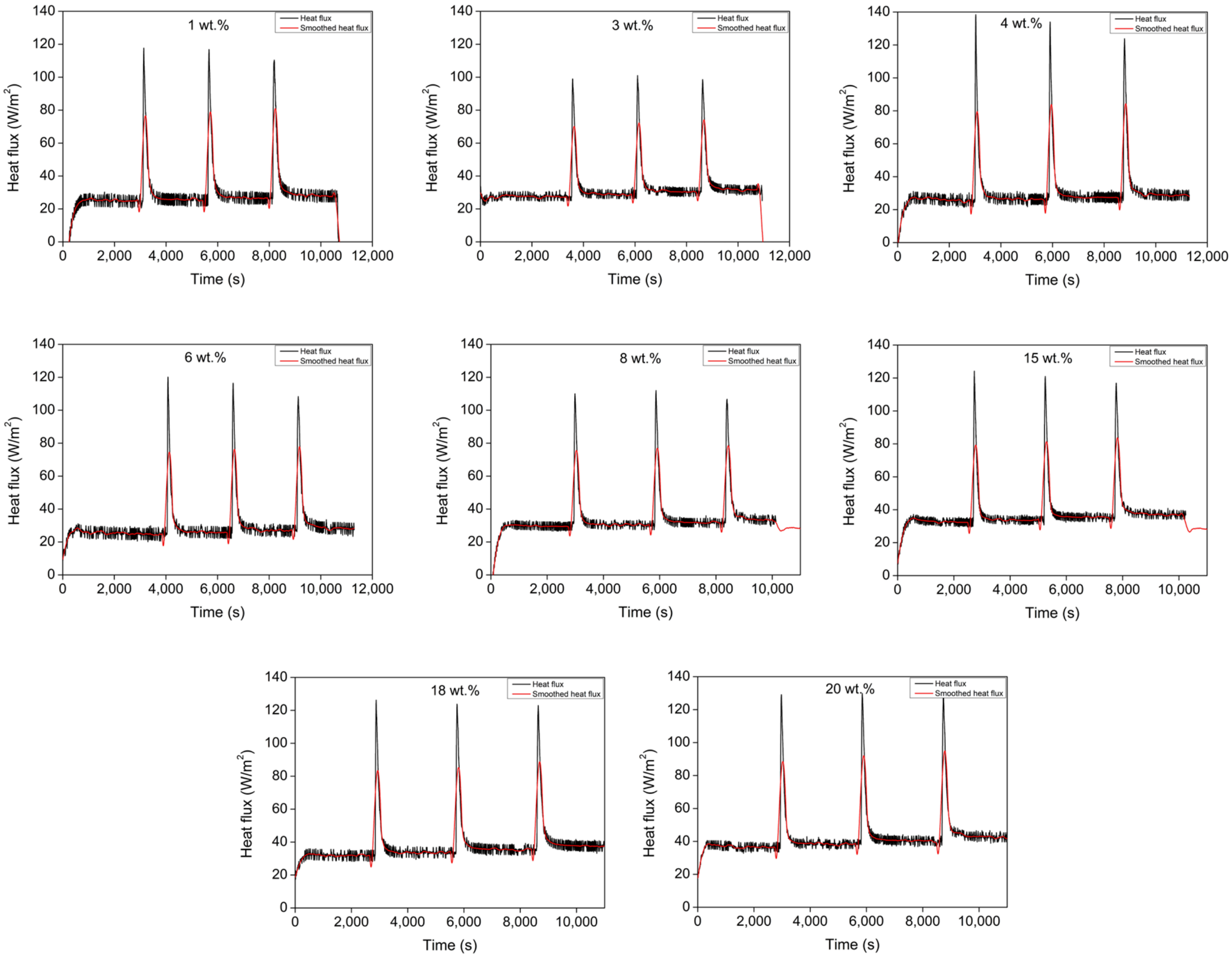

Figure S1. Heat flux obtained with an external sensor for different measurement temperatures under the stationary method.

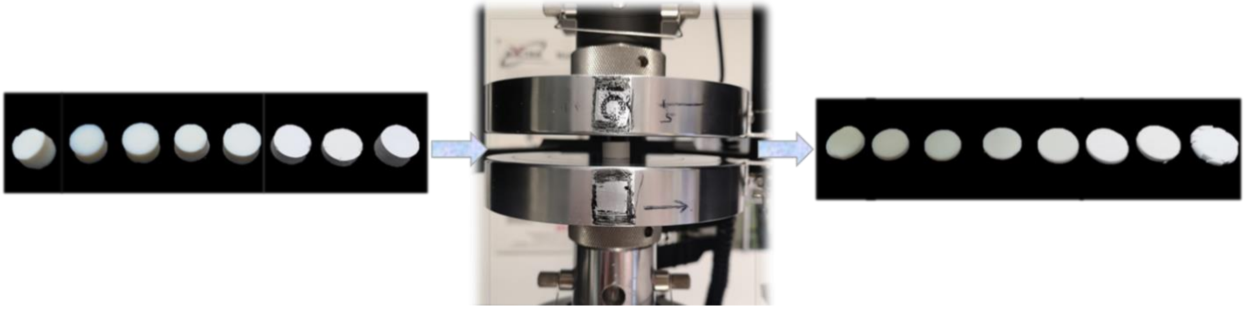

Figure S2. Aerogel PUR-PIR samples before (left) and after (right) the uniaxial compression tests.

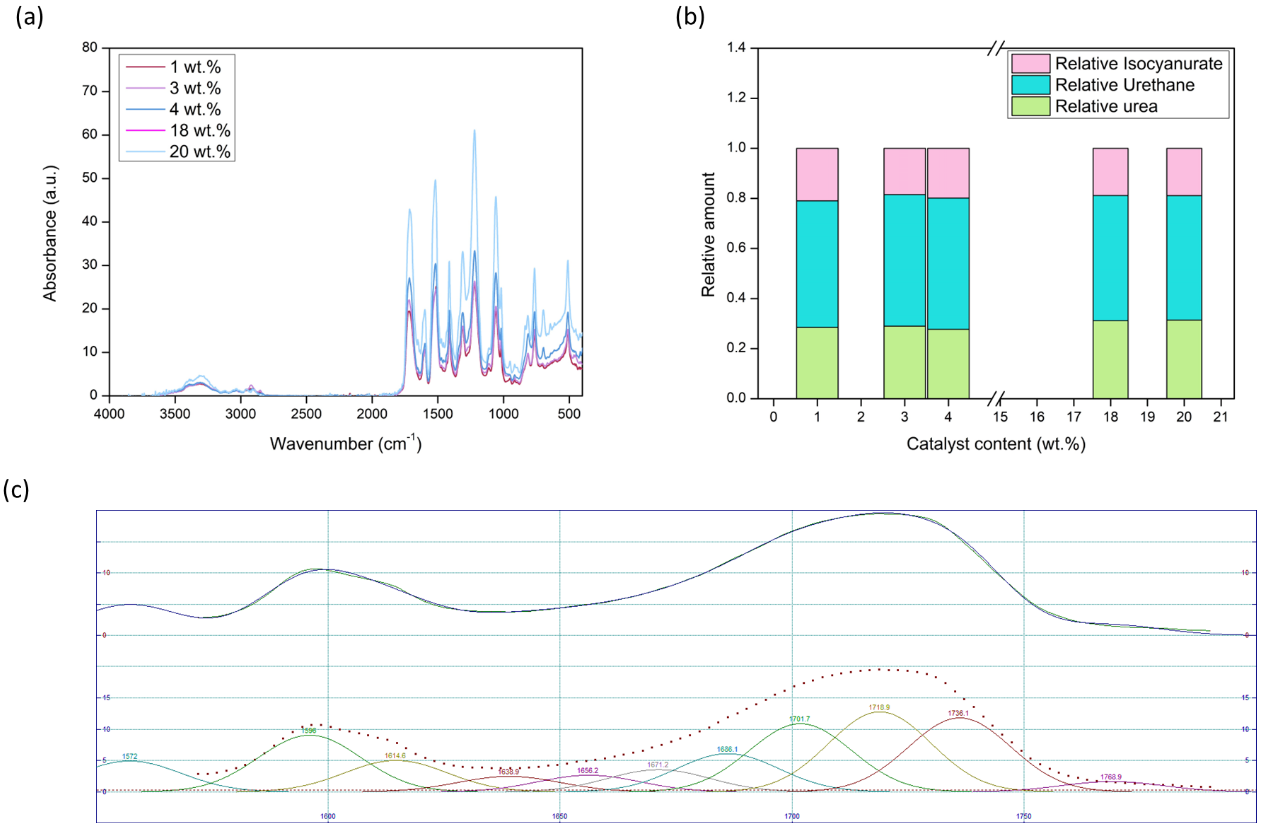

Figure S3. (**a**) FT-IR spectra of some PUR-PIR aerogels, (**b**) Relative area of each of the products of the amide I region for the different samples, (**c**) Example of an FT-IR spectrum deconvolution.

Table S1. Urea, urethane and isocyanurate total and relative areas.

	GENERAL RESULTS OF AMIDE I						
Sample	Total Area				Relative Area		
	Urea	Urethane	Isocyanurate	Carbonyl Region	Urea	Urethane	Isocyanurate
1wt	14.8	26.3	10.9	51.9	0.28	0.51	0.21
3 wt	19.6	35.6	12.5	67.6	0.29	0.53	0.18
4 wt	23.0	43.5	16.5	82.9	0.28	0.52	0.20
18 wt	39.4	63.3	23.9	126.6	0.31	0.50	0.19
20wt	39.7	63.0	23.9	126.6	0.31	0.50	0.19

Table S2. Elastic modulus value for all the aerogel samples.

Sample	Elastic modulus

	**(MPa)**
1 wt.%	6.32
3 wt.%	2.74
4 wt.%	0.55
6 wt.%	0.33
8 wt.%	0.24
15 wt.%	0.21
18 wt.%	0.10
20 wt.%	0.13

Table S3. Fitting parameters for the elastic modulus vs. the relative densities of the PUR-PIR aerogels.

**Model**	**Allometric1**		
Equation	y = a*x^b		
Reduced Chi-Sqr	0.23428		
Adj. R-Square	0.95161		
		Value	Standard Error
C	a	3.40×10^{9}	1.07×10^{10}
C	b	10.183	1.583
